# Supplementary material for: A Novel and Green Method for Preparing Highly Conductive PEDOT:PSS Films for Thermoelectric Energy Harvesting
Source: Polymers (Basel). 2024 Jan 18;16(2):266. doi: 10.3390/polym16020266 (PMC10820001; doi:10.3390/polym16020266)
Supplement: Supplementary file 1 [file polymers-16-00266-s001.zip › polymers-2772889-supplementary.pdf]

polymers

MDPI

Supporting Information

A Novel and Green Method for Preparing Highly Conductive PEDOT:PSS Films for Thermoelectric Energy Harvesting

Fuwei Liu [1,2,3,*,†], Luyao Gao [1,2,3,†], Jiajia Duan [1], Fuqun Li [1], Jingxian Li [1], Hongbing Ge [1], Zhiwei Cai [1], Huiying Li [1], Mengke Wang [1], Ruotong Lv [1] and Minrui Li [1]

[1] College of Physics and Electronic Engineering, Xinyang Normal University, Xinyang 464000, China
[2] Key Laboratory of Advanced Micro/Nano Functional Materials of Henan Province, Xinyang Normal University, Xinyang 464000, China
[3] Energy-Saving Building Materials Innovative Collaboration Center of Henan Province, Xinyang Normal University, Xinyang 464000, China
* Correspondence: liufuwei168@163.com
† These authors contributed equally to this work.

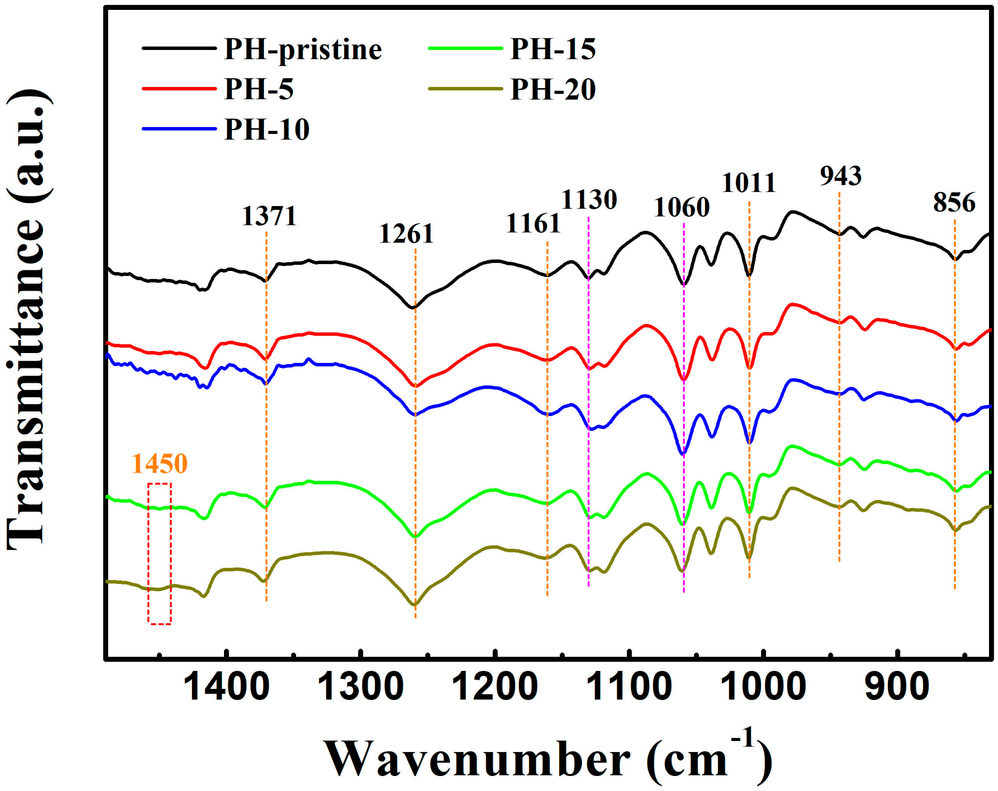
PH-pristine
PH-5
PH-10
PH-15
PH-20
1371
1261
1161
1130
1060
1011
943
856
1450
Transmittance (a.u.)
1400
1300
1200
1100
1000
900
Wavenumber (cm-1)

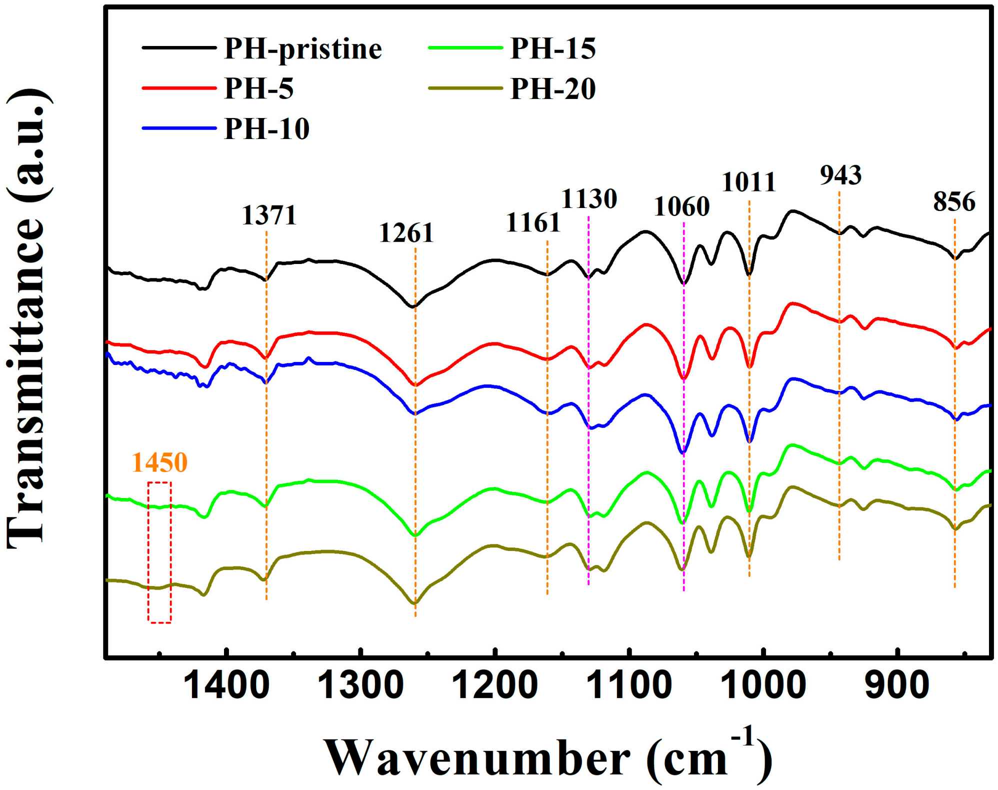
PH-pristine
PH-5
PH-10
PH-15
PH-20
1371
1261
1161
1130
1060
1011
943
856
1450
Transmittance (a.u.)
1400
1300
1200
1100
1000
900
Wavenumber (cm-1)

Figure S1. FTIR spectra of the PEDOT:PSS films before and after water washing treatment.

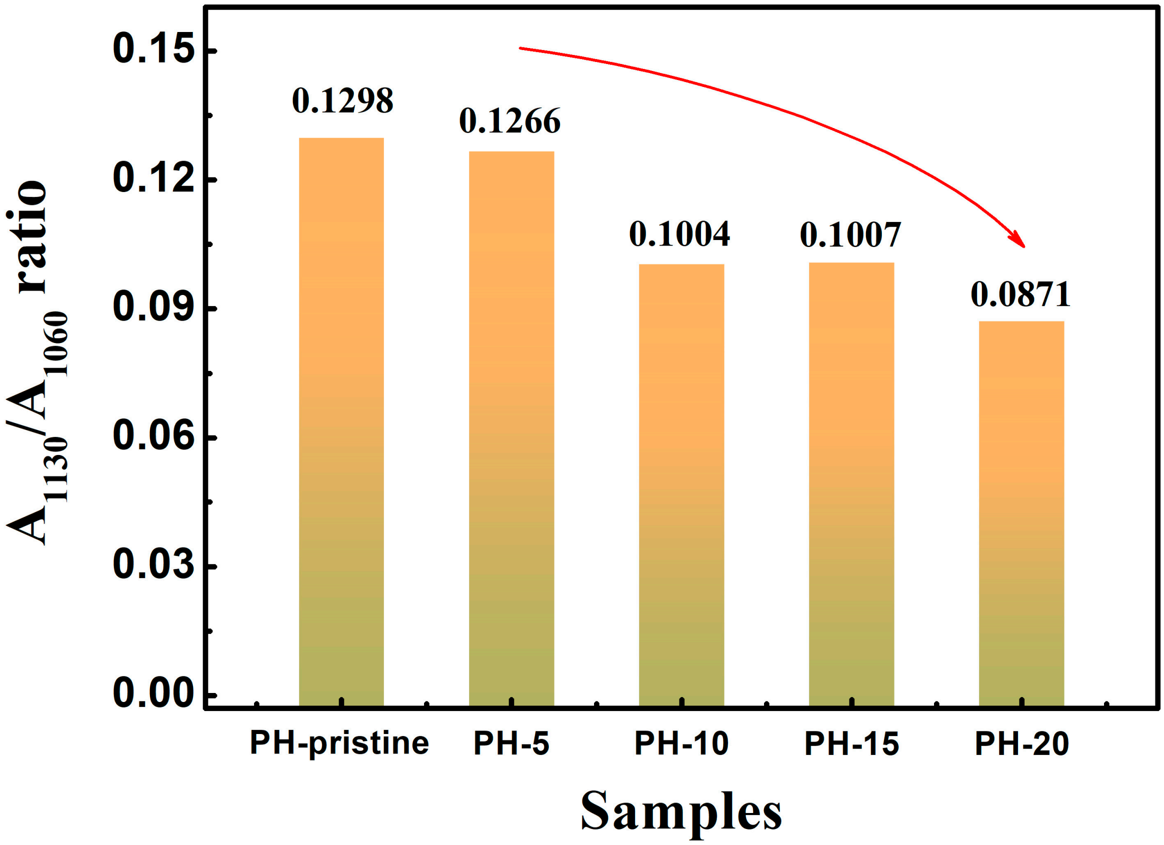

Figure S2. Variation of A_{1130}/A_{1060} ratio after different cycles of water washing treatment.

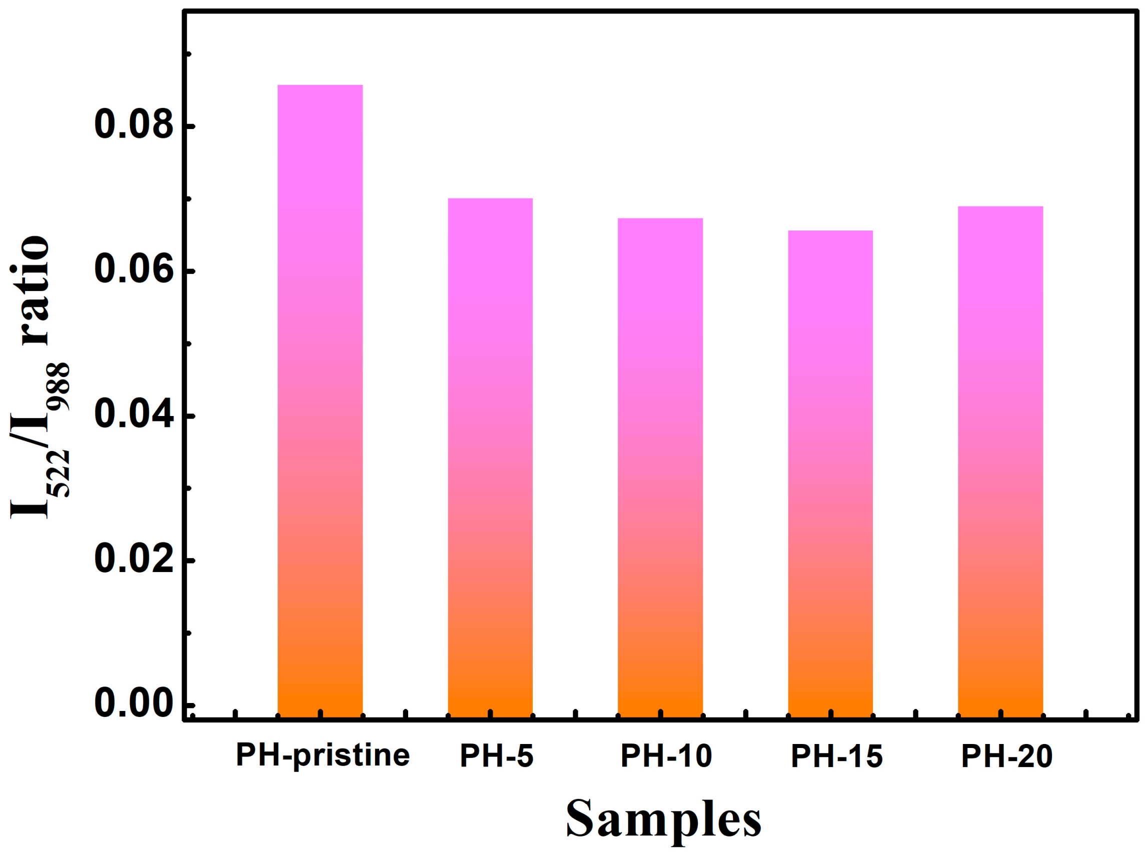

Figure S3. Variation of I_{522}/I_{988} ratio after different cycles of water washing treatment.

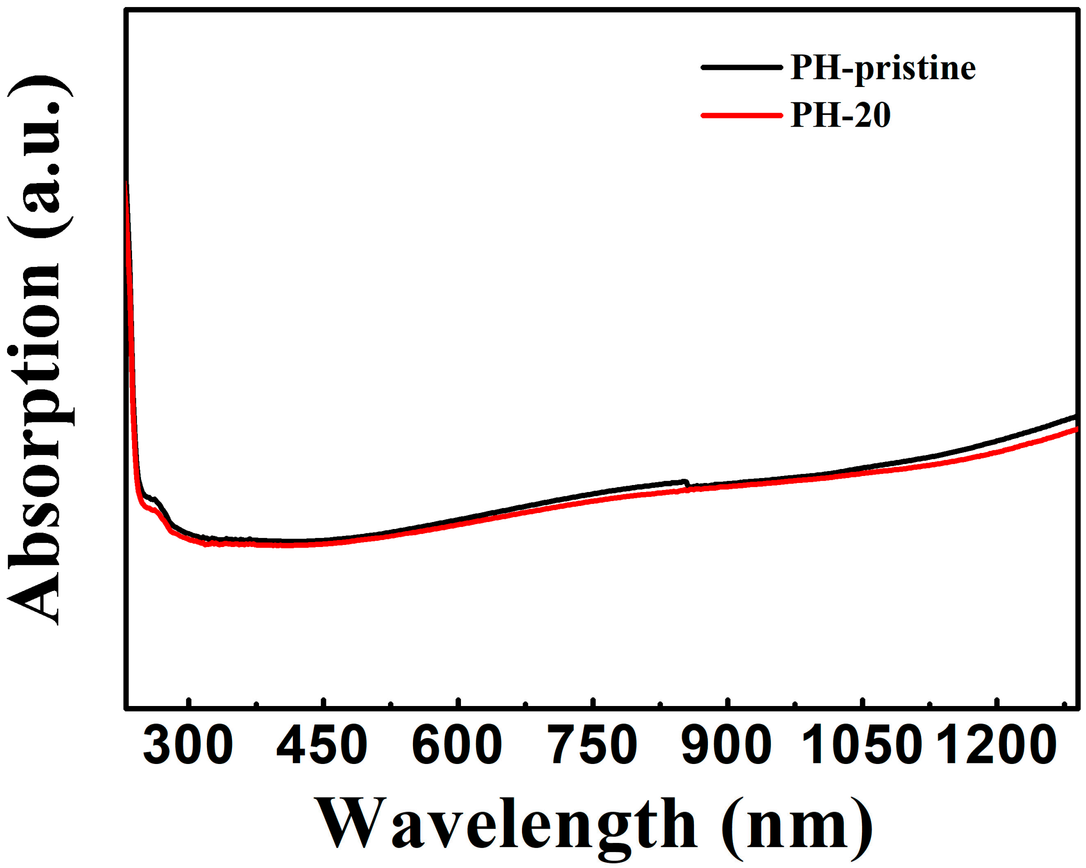

Figure S4. UV-Vis-NIR spectra before and after 20 cycles of water washing treatment.

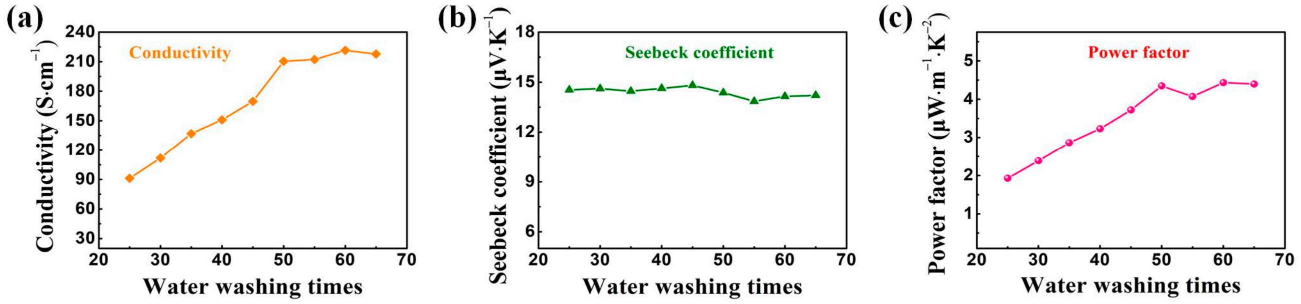

Figure S5. Variation of the TE performance of PEDOT:PSS film after further treatment with warm water: (a) conductivity, (b) Seebeck coefficients, and (c) power factor.

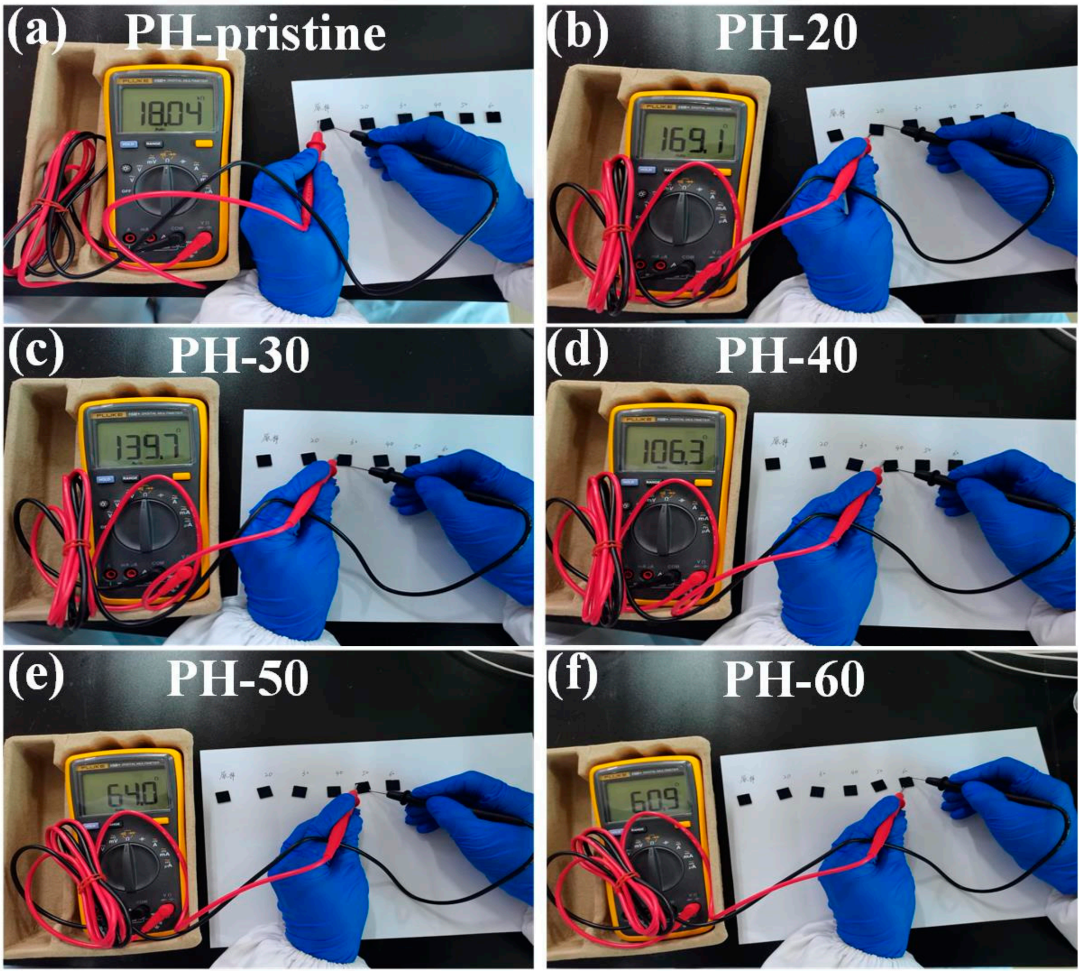

Figure S6. Variation of the PEDOT:PSS film resistance after water treatment.

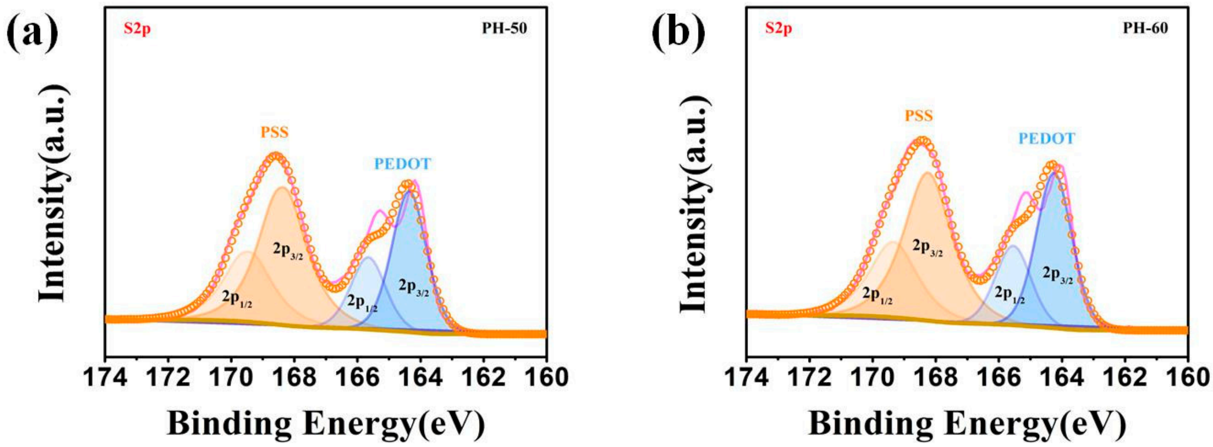

Figure S7. XPS spectra of PEDOT:PSS films with further warm water treatment: (a) S2p spectra of PEDOT:PSS film washed 50 times; (b) S2p spectra of PEDOT:PSS film washed 60 times.

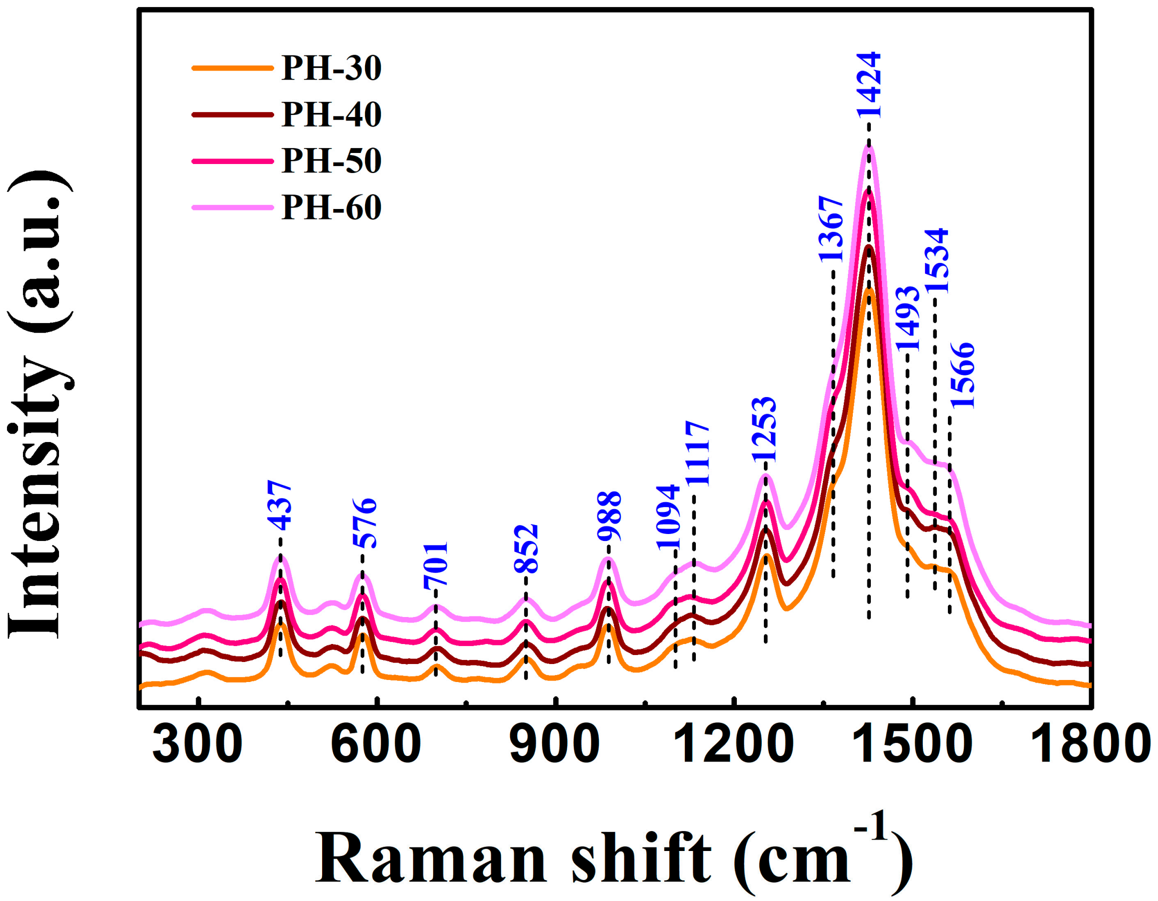

Figure S8. Raman spectra of PEDOT:PSS films after different times of warm water washing treatment.

Raman spectra of PEDOT:PSS after different times of warm water washing was depicted in Figure S8. As can be observed, the peak attributed to symmetric $C_α$=$C_β$(−O) stretching vibrations of the five-membered thiophene ring was further red-shifted to 1424 cm^{-1}, with the increase of the peak intensity, indicating more quinoid-dominated structure formed during warm water washing, thereby further enhancing of the charge-carrier mobility. Whereas, peaks centered at 1566 cm^{-1} and 1493 cm^{-1}, corresponding to asymmetric $C_α$=$C_β$ stretching, remained relatively stable after warm water treatment, which may be attributed to the relative stable doping level of the quinoid structure in PEDOT component [1]. XPS analysis was also performed to investigate the variation of the compositional changes in the PEDOT:PSS films during further warm water washing process. Calculations based on the changes in peak areas corresponding to PSS and PEDOT revealed that the PSS/PEDOT ratio decreased to 1.45 and 1.38 after 50 and 60 warm water washing cycles, respectively (Figure S7). Compared with those of the normal temperature water treated samples, the proportion declined slightly, suggesting that the PSS removal effect became relatively weak. Combined with Raman results, it is reasonable to infer that the increase of the conductivity during warm water treating is mainly induced by structural variation of PEDOT:PSS rather than its conformational changes.

References

1. Ely, F.; Matsumoto, A.; Zoetebier, B.; Peressinotto, V.S.; Hirata, M.K.; de Sousa, D.A.; Maciel, R. Handheld and automated ultrasonic spray deposition of conductive PEDOT:PSS films and their application in AC EL devices. *Organic Electronics* **2014**, *15*, 1062-1070, doi:10.1016/j.orgel.2014.02.022.
